# Greater magnocellular saccadic suppression in high versus low autistic tendency suggests a causal path to local perceptual style

**DOI:** 10.1098/rsos.150226

**Published:** 2015-12-16

**Authors:** David P. Crewther, Daniel Crewther, Stephanie Bevan, Melvyn A. Goodale, Sheila G. Crewther

**Affiliations:** 1 Centre for Human Psychopharmacology, Swinburne University of Technology, Melbourne, Australia; 2 La Trobe University, Melbourne, Australia; 3 Brain and Mind Institute, University of Western Ontario, London, Ontario, Canada

**Keywords:** autistic tendency, magnocellular, saccadic suppression, nonlinear visual evoked potential

## Abstract

Saccadic suppression—the reduction of visual sensitivity during rapid eye movements—has previously been proposed to reflect a specific suppression of the magnocellular visual system, with the initial neural site of that suppression at or prior to afferent visual information reaching striate cortex. Dysfunction in the magnocellular visual pathway has also been associated with perceptual and physiological anomalies in individuals with autism spectrum disorder or high autistic tendency, leading us to question whether saccadic suppression is altered in the broader autism phenotype. Here we show that individuals with high autistic tendency show greater saccadic suppression of low versus high spatial frequency gratings while those with low autistic tendency do not. In addition, those with high but not low autism spectrum quotient (AQ) demonstrated pre-cortical (35–45 ms) evoked potential differences (saccade versus fixation) to a large, low contrast, pseudo-randomly flashing bar. Both AQ groups showed similar differential visual evoked potential effects in later epochs (80–160 ms) at high contrast. Thus, the magnocellular theory of saccadic suppression appears untenable as a general description for the typically developing population. Our results also suggest that the bias towards local perceptual style reported in autism may be due to selective suppression of low spatial frequency information accompanying every saccadic eye movement.

## 1. Background

Saccadic suppression is the term for a loss of visual sensitivity that occurs around the time of a rapid eye movement, and was first reported around 1900 [1]. Its role in visual perception and its underlying neural mechanisms have continued to be explored over the past century. Early accounts of the neural mechanism underlying the phenomenon [2] invoked extra-retinal sensing, through efference copy of the oculomotor impulses. Single cell studies in primate indicate that the activity of some parietal cortical neurons [3] correlates with a shift in attention to the new location approximately 50 ms prior to the actual eye movement. The multiple representations of locations and objects of interest in parietal cortex are also thought to provide the means for the smooth transition from retinotopic to egocentric coordinates [4] in preparation for visually guided action.

In 1994, Burr *et al.* [5] argued that saccadic suppression is selective for information conveyed by the magnocellular rather than by the parvocellular visual pathway. They based this finding on a detailed series of psychophysical experiments with three participants in which thresholds for visibility of horizontal gratings, varying either in luminance or colour, were measured over a range of spatial frequencies. For monochromatic gratings, the degree of suppression (ratio of threshold for presentation during large saccadic eye movements to that during free viewing) varies from a value of approximately 10 at low spatial frequencies (0.02 cpd) to close to 1 for high spatial frequencies (3 cpd). For colour modulated gratings, however, there was little variation in suppression, maintaining values close to 1.0 across the same range of spatial frequencies. Thus, on the basis of the properties of primate magno- and parvocellular neurons (reviewed in [6]), Burr *et al*. [5] surmised that suppression was confined to the colour insensitive magnocellular visual stream. An early site for the neural locus of saccadic suppression between retina and visual cortex has been proposed on the basis of a motion psychophysics study [7] and of a transcranial magnetic stimulation (TMS) study showing that retinally induced phosphenes suffer saccadic suppression while those cortically generated do not [8].

Deficits in magnocellular/dorsal stream processing have been proposed as an explanation for perceptual abnormalities in autism [9–14]. Individuals diagnosed with autism spectrum disorders (ASD) often show superior perception of local detail [15], with a bias towards more local elements at the expense of the global components of an image [16] (though see [17]). Atypical sensory perception in ASD has been linked to the poor processing of eye gaze and facial expressions, as well as less efficient motion processing [10,11]. The majority of motion coherence and biological motion studies in autism and the broader autistic phenotype show raised thresholds (reviewed [18]), although there are still questions regarding the cortical level at which the magnocellular/motion processing system experiences difficulty [12,14,19]. Taken together, these findings provide a compelling argument for examining saccadic suppression across the autistic spectrum.

The differentiation between the reported effects of saccadic suppression on magno- and parvocellular properties [5] suggests the use of electrophysiological measures capable of differentiating magno- and parvocellular afferents. One such visual evoked potential (VEP) approach uses separate stimuli biased towards magno- (M) and parvocellular (P) properties [20–23]. An alternative method, based on nonlinear analysis of the multifocal VEP, generates simultaneous estimates of M and P systems from VEP nonlinearities generated under rapid pseudorandom stimulation [24,25]. These contributions from achromatic stimulation show an M-derived nonlinearity with high gain at low stimulus contrast and saturation at high contrast with the P-derived nonlinearity showing lower contrast gain without saturation at high contrast. The M contribution has a 25–30 ms latency ‘magnocellular advantage’ [26]. Recent nonlinear VEP kernel analysis of groups high and low in autistic tendency demonstrated significant differences in magnocellular function [27,28], with weaker first-order response at low contrast and a delayed completion of the second-order M component at high contrast. P-driven components were comparable for individuals high and low on the autism spectrum quotient (AQ) [29]. Findings of impaired magnocellular function in high autistic tendency raise the question of what differences in saccadic suppression of magnocellular function we would expect to find between high AQ and low AQ groups. The status of autistic tendency in prior saccadic suppression investigations has not been reported, and previous studies have not been sufficiently powered [5,7,8,30–34], to detect any such differences.

Thus, in this study, we aimed to psychophysically measure saccadic suppression in typically developing non-clinical populations scoring either high or low on the AQ scale, using achromatic gratings of low (0.3 cpd) and high (2.0 cpd) spatial frequency. These spatial frequencies were chosen on the basis of the stimuli used by Burr *et al.* [5]. Chromatic gratings were not used, given the reported lack of differences in visual sensitivity to chromatic gratings presented within or between saccadic eye movements [5]. We predicted that saccadic suppression of the M-pathway would be greater in those with high AQ than in those with lower autistic tendency, given the literature outlined above, describing a weakness or deficit in magnocellular processing in those with high autistic tendency.

We also aimed to assess the effects of saccades on the multifocal VEP by comparing conditions where VEPs were recorded in a fixation condition and during a condition requiring frequent self-generated saccade execution. We predicted that, apart from the known alteration in VEP waveforms in the second-order response associated with magnocellular processing in those with high AQ, we would observe both short and long latency VEP differences under saccade compared with fixation conditions for those with high AQ scores compared with those with low AQ. Any such differences would be expected to reflect magnocellular contributions to afferent input for cortical processing, and to top-down facilitation [35] of recognition and perception.

## 2. Material and methods

Sixty-two participants low and high in autistic tendency were recruited via an online version (Opinio) of the AQ scale [29]. Of those who responded, 13 high AQ (AQ score≥19) and 14 low AQ (AQ score≤12) participants underwent psychophysical testing for saccadic suppression. Fourteen high AQ and 14 low AQ participants also underwent measurement of nonlinear VEPs under fixation and saccade conditions. The number tested in total in each AQ group, the mean score with standard deviation and the range of AQ scores are shown in table 1.

**Table 1.** AQ scores for high and low AQ groups drawn from the typically developing adult population.

AQ group	number	mean±s.d.	range
low AQ	14	7.7±3.1	3–12
high AQ	18	25.4±4.0	20–32

### 2.1 Psychophysics

Grating stimuli were generated in Vpixx (www.vpixx.com) to appear on the screen (CRT monitor, mean luminance 60 cd m^−2^) for two monitor frames (27 ms, using a 75 Hz refresh rate) upon the receipt of a trigger from the eye movement system (Skalar, IR limbal eye tracker, 1 kHz) when the participant made a saccadic eye movement from a point on the left of the screen to one which appeared on the right, one frame after the left dot was extinguished.

In the saccade condition, the grating was presented in one of four locations between the two fixation points (two above, two below the line joining the two points) immediately on sensing of the initiation of the saccade. In the delay condition, a delay of 170 ms after saccade initiation was executed prior to stimulus presentation, allowing for recovery of visual sensitivity after the saccade completion [36]. Grating contrast was controlled via a four alternate forced choice (4AFC) protocol for the spatial location of the presented grating.

### 2.2 Nonlinear visual evoked potential

**Figure 1.**
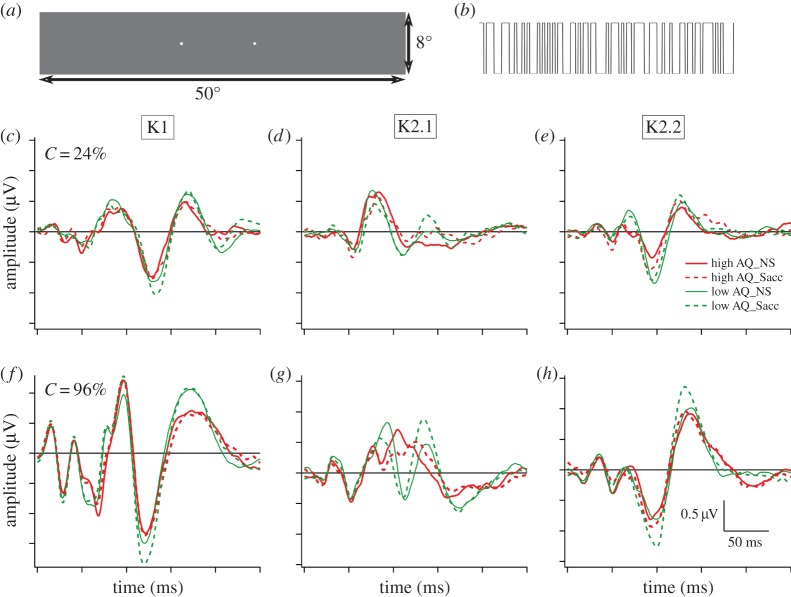
VEP stimulus—a uniform rectangle (50°×8°) (*a*) was alternated in a pseudo random fashion (*b*) between two luminance levels. Two points 10° apart were used for either fixation or as saccade targets. First- and second-order kernel responses were recorded at low contrast (*c*–*e*, 24%) and high contrast (*f*–*h*, 96%). Solid line traces indicate VEPs recorded during fixation conditions while dotted lines indicate VEP recorded during rapid and frequent saccadic eye movements (not coordinated with the pseudorandom binary focal stimuli). Red lines indicate mean responses from the high AQ group; green lines indicate responses from the low AQ group.

Nonlinear visual evoked potentials were measured using a VERIS system (EDI, San Mateo, v. 3) with a customized stimulus comprising a single rectangular patch subtending 50° horizontally by 8° vertically. Two small fixation dots were placed on the screen 10° apart. The luminance of the patch fluctuated between two levels, under the control of a pseudorandom binary *m*-sequence (*m*=14; figure 1*a*,*b*). Each 4 min sequence was divided into 8×30 s epochs. In one condition, the participant maintained fixation on either one of the dots for 30 s. In the second condition, the participant made rapid saccades at a rate of approximately 2 Hz between the dots for the duration of a 30 s epoch. The fixation and saccade condition epochs were interleaved. Stimuli were binary diffuse luminance patches with temporal contrast of either 24% or 96% around the mean luminance of 50 cd m^−2^. Wiener kernel analysis (built into the VERIS system) resulted in the extraction of waves for the first-order (K1) and the first two slices of the second-order kernel (K2.1, K2.2). A brief guide to nonlinearities and the Wiener kernel expansion can be found in the electronic supplementary material.

## 3. Results

### 3.1 Psychophysics

**Figure 2.**
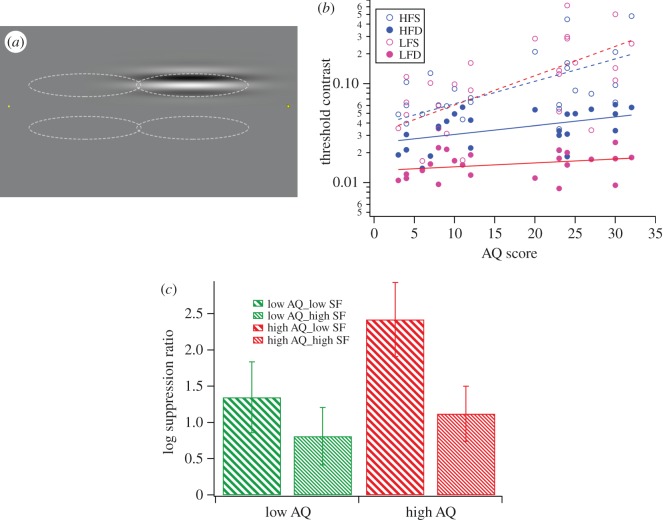
(*a*) A 40° saccade caused the immediate or delayed presentation of a Gabor patch containing either a low (0.2 cpd) or high (2.0 cpd) spatial frequency grating in one of four locations (indicated by dotted lines), using a 4AFC protocol. (*b*) Contrast thresholds measured for individuals for presentation during a saccade (closed circles) compared with delayed (open circles)—pink, low and blue, high spatial frequency; regression lines are dotted for saccade conditions and solid for delay conditions. (*c*) Log suppression ratios with confidence intervals are shown for the groups of high AQ (red) and low AQ (green) for low spatial frequency (coarse pattern) and high spatial frequency (fine pattern).

Contrast thresholds for both immediate and delayed presentation of both high and low spatial frequency gratings were established using the VPEST (Parameter Estimation by Sequential Testing) routine built into the Vpixx software. This uses a maximum-likelihood estimation of threshold based on performance in all trials up to that point, with the contrast level set at that estimate for the next trial. The high AQ group showed suppression for both high and low spatial frequency stimuli, with much greater suppression for gratings of low versus high spatial frequency. This pattern of suppression is reminiscent of saccadic suppression as described by Burr *et al*. [5] (figure 2). The low AQ group showed more equal suppression for high and low spatial frequency gratings, and suppression of low spatial frequency gratings was markedly reduced compared with that for the high AQ group.

Following removal of one outlier (one low AQ participant identified by JMP statistics software (SAS Institute Inc.) with high spatial frequency results greater than 2.5 s.d. from the mean), statistical analysis (repeated measures ANOVA, 13 high AQ, 13 low AQ) showed significant main effects of spatial frequency (*F*_1,22_=30.06,*p*<0.0001) and AQ group (*F*_1,22_=7.362,*p*=0.013), as well as a significant interaction of spatial frequency × AQ group (*F*_1,22_=5.66,*p*=0.027). Post hoc *t*-tests indicated that while suppression was significant between groups at low spatial frequency (*t*=3.15,*p*=0.005), there was no significant difference in suppression at high spatial frequency.

### 3.2 Electrophysiology

The current study used a much larger stimulus area than in previous multifocal mapping studies, in order to keep the fovea well within its bounds, even when making 10° saccadic eye movements. The temporal structure of the VEP conformed with previous reports [27,28] with similar peaks in the various kernel slices. However, very short latency features emerged that have not been previously reported.

#### 3.2.1 First-order kernel K1

Apart from the large and recognizable N65–P95–N125–P175 peak structure, the first-order kernel responses recorded at high luminance contrast (figure 1*f*) showed novel, early and well-defined peaks at latencies less than 45 ms. These are not normally seen in small stimulus patch size (typically 2°–4°) multifocal VEP studies [25,28]. The generators of such early responses are likely to lie in the lateral geniculate nucleus (LGN). Early LGN contributions to the flash-evoked cortical VEP have been reported in primate recordings [37,38]. A sudden increase in variance of the VEP signals at approximately 45 ms is likely to reflect transition from LGN to cortical contributions to the VEP [20,39]. This timing is also consistent with the first V1 neural spikes in non-human primates (typically 35 ms latency [40]). The extra 5–10 ms in humans is presumably a conduction time difference due to the relative sizes of monkey and human brains.

In terms of eye movement conditions, the first-order responses were generally similar in waveform across both AQ groups and saccade/fixation conditions, with some suggestion of an enhanced N125 peak for the low AQ group under the saccade condition. There were minor AQ-related deviations for high contrast stimulation at approximately 50 and 70 ms (figure 1*f*).

#### 3.2.2 Second-order kernel first slice K2.1

The second-order first slice response amplitudes were already close to saturation at the lower 24% contrast (as would be consistent with magnocellular generation and with previous nonlinear VEP recordings [28]).

In the first slice of the second-order kernel (K2.1) at low contrast, there was a saccade-dependent deviation in the mean waveforms occurring at approximately 70 ms for both AQ groups (see figure 1*d*). At high contrast, the greatest AQ-related anomalies in waveform occurred in the first slice of the second-order kernel (K2.1) (figure 1*g*). The main positivity for the high AQ group showed a marked notch at approximately 70 ms (reminiscent of an earlier observation [27]) with a maximum occurring at approximately 105 ms in the high AQ fixation condition. Under the saccade condition (figure 1*g*, 96% contrast), the positive peak for the high AQ group was markedly suppressed with the peak extended to longer latencies (red dashed line—peak 125 ms). By comparison, the low AQ K2.1 group responses (figure 1*g*) were well described by two positive peaks at 90 ms and 130 ms with a conspicuous shift in weighting of the amplitudes to the longer latency peak under saccade compared with fixation conditions. Repeated measures analysis of these peak amplitudes showed strong effects of eye movement condition (*p*<0.005 for both high and low contrast figure 1*d*,*g*) and eye movement × peak interactions (*C*=96%:*F*=40.02, *p*<0.0005; *C*=24%:*F*=40.12, *p*<0.0005) with a marginal effect of AQ group (*F*=3.59, *p*=0.069).

#### 3.2.3 Second-order kernel second slice K2.2

The major peak of the second-order, second slice response showed strong growth from low to high contrast (consistent with generation by the parvocellular pathway). The K2.2 response showed an effect of fixation versus saccade on the N95–P130 amplitude (*F*=16.2, *p*<0.0005) for high-contrast stimulation (see figure 1*h*), but any effects of AQ group were not significant.

### 3.3 Differential effect of saccades on visual evoked potential

**Figure 3.**
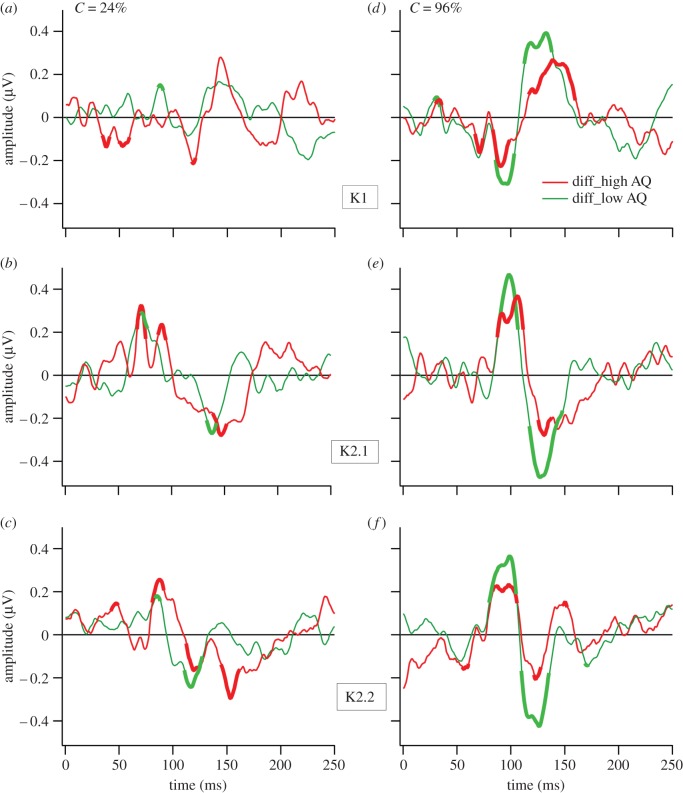
Saccadic suppression of VEP responses shown as difference curves (fixation–saccade) for low contrast (*a*–*c*) and high contrast (*d*–*f*). Portions of the traces where the difference curves exceed the 95% confidence interval departure from zero are shown with thickened lines. (*a*) The high AQ group shows evidence of a very early significant saccade related difference. (*b*) Both AQ groups showed a K2.1 difference approximately 75 ms latency. (*c*) At high contrast an early brief effect of saccades is found in both AQ groups. (*d*–*f*) The saccade-generated differences at high contrast are remarkably similar in waveform and timing across K1, K2.1 and K2.2 waveforms, with the high AQ group showing generally lower amplitude curves.

In focusing on the question of saccadic suppression in high versus low AQ, difference waves (fixation—saccade) were calculated (figure 3). Intervals for which these difference waveforms departed from zero by more than 1 confidence interval (95%) are portrayed by a thickening of the particular trace. For the high AQ group low contrast recording (figure 3*a*), an early (36–42 ms) epoch showed significant effect of saccades—just prior to the time of cortical activation [20,28]. This early effect was not observed in the low AQ group, but was observed for both AQ groups at high contrast (figure 3*d*).

Inspection of figure 3*b* shows early cortical saccade-related effects in the second-order first slice (K2.1), clearly visible at low contrast for both AQ groups. In addition, a third period of saccade-related effects for the difference waves can be seen in the latency range 80–160 ms. These longer latency difference effects are quite uniform in terms of waveform across kernels K1, K2.1 and K2.2 (figure 3*d*–*f*) in the high contrast recordings, apart from a difference in amplitude across AQ groups. Also, there is a strong contrast dependence, with the difference curve amplitudes much larger at high compared with low contrast. A check against figure 1 indicates that in the first-order VEP, the waves are generally greater in amplitude during saccades than under fixation conditions.

The psychophysical and physiological datasets can be found in the electronic supplementary material.

## 4. Discussion

The findings of the current study make it clear that the previous arguments for a selective suppression of the magnocellular pathway in the typically developing population under saccadic conditions should be re-examined. This is because significantly greater perceptual suppression for low compared with high spatial frequency stimuli only occurs for those with high autistic tendency but not for those with low autistic tendency. All the participants in this study were recruited from the typically developing population. In addition, the percentage of the typically developing population with AQ scores of 12 and below (the criterion for our low AQ group) is over 23%, based on a recent large sample of 450 [41]. By comparison, the literature on saccadic suppression comprises studies with small population samples that may or may not cover the variation of autistic tendency in the population. Indeed, if such participants were recruited from scientists, engineers or students studying these courses, then higher AQ scores than the population mean would be expected [29].

The VEP recordings add weight to the notion that saccadic suppression occurs relatively early with respect to the time of cortical activation (starting at approximately 45 ms) [20,28]. However, here again AQ group differences were found—at low contrast, pre-cortical suppression was limited to the high AQ group [37]. However, two other VEP epochs were also associated with significant saccade-related activity differences. The first of these occurred in the VEP second-order nonlinear kernel peak (approximately 75 ms) associated with magnocellular function [25,28]. Curiously, despite the manifest differences between the VEPs of the high and low AQ groups (particularly figure 1*f*), the effects of saccades as measured by the mean difference waves (cf. figure 3*d*–*f*) were surprisingly similar. For both high and low autistic tendency groups, saccade-related VEP differences were measured in the latency range of 80–160 ms. Remarkably, the second-order difference responses appear to be tightly aligned to the first-order difference waves (particularly at high contrast—figure 3*d*–*f*), suggesting that during this period, occipital cortex is differentially responsive to visual stimulation. Indeed, the larger amplitudes recorded under saccade versus fixation conditions indicate the possibility of an increase in excitability/decrease in inhibition as a likely response to saccades. Thus, area V1 is not suppressed in terms of physiological response, at least for flashed diffuse stimuli. Also, the finding that this later (80–160 ms) fixation versus saccade VEP difference is contrast dependent, with much larger fixation minus saccade differences at high compared with low contrast stimulation, is indicative of parvocellular influence. These later saccade-dependent VEP effects may relate perceptually to saccadic omission [42], the phenomenon whereby visual stimuli at seemingly suprathreshold contrasts are ‘omitted’ from perceptual report during saccades, rather than saccadic suppression that is traditionally measured as a contrast threshold phenomenon.

In conclusion, we have demonstrated that saccadic suppression has different effects on the suppression of low- and high-spatial frequency stimuli in typically developing populations categorized by the AQ scale into groups high and low in autistic tendency. What are the implications for individual differences in perception and perceptual development? In daily life, individuals continually make eye, head and body movements as they pass through a perceptually stable visual environment. Indeed, even when fixating, we make two or three small saccades every second [43], and such microsaccades have been shown to cause physiological suppression as well as behavioural effects in the primate visual system [44]. Thus, if saccades have the tendency to inhibit low spatial frequencies selectively in those with high autistic tendency, then the resulting perceptual state would be continually biased towards higher spatial frequencies, equating to more visual detail and possibly more local compared with global information. This could be construed as a more local attentional style as described in DSM-5 for those with ASD. In addition, the contribution of rapid magnocellular projections from visual to orbitofrontal and then to inferotemporal cortex to facilitate top-down recognition processes [35] is likely to be differently affected during saccades by those with high compared with those with low autistic tendency, further impacting on perceptual style. Hence we suggest that the local perceptual style exhibited by those with high autistic tendency may be the result of this selective inhibition of low spatial frequencies during saccades.

## Supplementary Material

SupplementaryInformation_WienerKernels.docx

## Supplementary Material

SaccSuppPsychophys2.xlsx This spreadsheet contains column headings AQscore, AQgroup, HFS, HFD, LFS, LFD, HFratio, LFratio, LogHFr,LogLFr, where HF stands for high frequency, LF = low frequency, S=saccade, D=delay.

## Supplementary Material

“nonlinearVEP_LowAQ.xlsx” contains similar data for the 13 Low AQ participants.

## Supplementary Material

“nonlinearVEP_HighAQ.xlsx” contains the evoked potential data for first order kernel:K1, first slice of second order kernel K2.1 and second slice of second order kernel K2.2 with separate columns for each participant. Data derived for saccade condition are labelled with “S” while data recorded in the Fixation conditions are labelled “NS”. Two contrast levels C24 = 24% contrast and C100 = 100% contrast.

## Data accessibility

The datasets supporting this article have been uploaded as part of the electronic supplementary material.
