# Supplementary material for: Greater magnocellular saccadic suppression in high versus low autistic tendency suggests a causal path to local perceptual style
Source: R Soc Open Sci. 2015 Dec 16;2(12):150226. doi: 10.1098/rsos.150226 (PMC4807440; doi:10.1098/rsos.150226)
Supplement: SupplementaryInformation_WienerKernels.docx [file rsos150226supp1.docx]

**Supplementary Information**

**A brief guide to nonlinearities and Wiener kernels**

The Wiener kernel decomposition (Wiener, 1958) of the temporal response for a system is an orthogonal expansion for nonlinear functionals closely related to the Volterra series, in which output of the nonlinear system depends on the input to the system at other times. This is particularly useful for analyzing neural systems where there is a neural memory, such that the response depends on the immediate past history of stimulation. While conceptualized in the late 1950s, progress only really occurred following the computer explosion of the 1980s. The early work of Sutter (1975) using pseudorandom spatiotemporal pattern stimuli, emerged as the VERIS system (EDI, San Matero, USA) for multi-focal measurement of the electroretinogram (mfERG). The method relies on deterministic white noise for orthogonality of kernels and to this end, m-sequences have been one of the most popular methods employed. An m-sequence produces all possible binary numbers m bits long (except for zero). Thus an m=14 sequence results in a total of 2^14^ – 1 binary states, which when delivered through a 75Hz stimulus monitor, changing state every frame, results in a stimulus sequence of length of 3 min 38 s.

Interpretation of the kernels:

The first order kernel is one half of the difference between the sum of the responses to state 1 (e.g. Stimulus = White) and responses to state 2 (e.g. Stimulus = Black). Thus if the stimulus is a pseudorandom series of black and white patches, the first order kernel $K1={1/2(R}_{W}-R_{B})$, where $R_{W}$is the sum of response to White and $R_{B}$is the sum of responses to Black. Thus it is difference of two event related potentials and is the impulse response of the system (Sutter, 1992).

The second order kernel takes account of the history of stimulation, and where stimulation using a computer monitor is limited by the frame rate, is thus a series of slices with the slice number representing an “interaction time”. Intuitively, it could be regarded as measuring the degree of recovery of the neural system to stimulation that occurred $n$ frames prior, where $n$ is the slice number. While theoretically there could be an infinite number of kernel contributions to the nonlinear focal VEP, under conventional stimulus conditions (frame rates of 60 or 75 Hz), most power is found in the first order and the first two slices of the second order response.

Thus the second order first slice is $K2.1=1/4(R_{WW}+R_{BB}-R_{WB}-R_{BW})$, where $R_{WW}$is the response to two White frames in a row, and $R_{BB}$is the response to two Black frames, and so on. The second slice of the second order kernel $K2.2$ has the same form as $K2.1$, however the reference stimulus is one frame further back with frames of either polarity in between. This can be seen diagrammatically in (Sutter, 2000).

While Sutter (Sutter, 2000, Sutter, 1992) felt that the higher order terms in the Wiener kernel expansion for the ERG did not reflect different neural contributions, Klistorner et al (1997) and Jackson et al (2013) show that the major peaks of the first and second order kernels of the nonlinear VEP show differential contrast gain and saturation properties that conform well with the known properties of primate magno and parvocellular visual pathways.

**References**

Jackson, B.L., Blackwood, E.M., Blum, J., Carruthers, S.P., Nemorin, S., Pryor, B.A., Sceneay, S.D., Bevan, S., & Crewther, D.P. (2013). Magno- and Parvocellular Contrast Responses in Varying Degrees of Autistic Trait. PLoS One, 8 (6), e66797.

Klistorner, A., Crewther, D.P., & Crewther, S.G. (1997). Separate magnocellular and parvocellular contributions from temporal analysis of the multifocal VEP. Vision Res, 37 (15), 2161-2169.

Sutter, E. (1975). A revised conception of visual receptive fields based on pseudorandom spatio-temporal pattern stimuli. 1st symposium on testing and identification of nonlinear systems (pp. 353-365). California Institute of Technology.

Sutter, E. (2000). The interpretation of multifocal binary kernels. Doc Ophthalmol, 100 (2-3), 49-75.

Sutter, E.E. (1992). A deterministic approach to nonlinear systems analysis. In: R. Pinter, & B. Naber (Eds.), Nonlinear Vision (pp. 171-220). Boca Raton, FL: CRC press.

Wiener, N. (1958). Nonlinear Problems in Random Theory. (Cambridge, Massachusets: Technology Press.
